# Lateral and apical root resorption in teeth orthodontically moved into edentulous ridge areas

**DOI:** 10.1590/2177-6709.25.5.024-029.oar

**Published:** 2020

**Authors:** Adilson Luiz Ramos, Rodrigo Lorenzi Poluha, Pablo Guilherme, Gabriel Araújo Khoury, Joao Marcos Pedro Rosa

**Affiliations:** 1 Universidade Estadual de Maringá, Departamento de Odontologia (Maringá/PR, Brazil).; 2 Private practice (Guaíra/PR, Brazil).; 3 Private practice (Maringá/PR,Brazil).

**Keywords:** Tooth movement, External root resorption, Edentulous alveolar ridge

## Abstract

**Objective:**

The present study aimed at comparing the external lateral root resorption (ELRR) and external apical root resorption (EARR) between teeth moved through the atrophic edentulous ridge and those undergoing the usual orthodontic movement.

**Methods:**

Fifty-four premolars were evaluated, where 27 of them had been moved toward the edentulous ridge (Group 1) and 27 from the same patient, had not been translated, which comprised the control group (Group 2). ELRR was evaluated by 0-3 scores and EARR was evaluated by 0-4 scores, before and after movement. Measurements were compared by Kruskal-Wallis and Student-Newman-Keuls tests.

**Results:**

ELRR increased statistically only in the Group 1 (*p*< 0.05). After orthodontic treatment, it was observed that almost 56% (n = 15) of teeth in Group 1 presented scores 2 and 3, while Group 2 presented scores 2 and 3 in about 11% (n= 3) of the teeth. EARR increased in both groups after orthodontic movement, however, statistically analyses showed no significant differences between groups (*p*> 0.05).

**Conclusions:**

Orthodontic movement into the atrophic edentulous ridge is subject to a greater lateral external root resorption.

## RESUMO

**Objetivo:**

O presente estudo teve como objetivo comparar a reabsorção radicular lateral externa (RRLE) e a reabsorção radicular apical externa (RRAE) em dentes movimentados sobre o rebordo edêntulo atrófico e naqueles submetidos à movimentação ortodôntica usual.

**Métodos:**

Foram avaliados 54 pré-molares, sendo 27 deles movimentados em direção ao rebordo edêntulo (Grupo 1) e 27 (dos mesmos pacientes) não transladados, que compuseram o grupo controle (Grupo 2). A RRLE foi avaliada por meio de escores de 0 a 3, e a RRAE foi avaliada por meio de escores de 0 a 4, antes e depois do tratamento. As medidas foram comparadas por meio dos testes de Kruskal-Wallis e de Student-Newman-Keuls.

**Resultados:**

O escore da RRLE aumentou estatisticamente apenas no Grupo 1 (*p*< 0,05). Após o tratamento ortodôntico, observou-se que quase 56% (n = 15) dos dentes do Grupo 1 apresentaram escores 2 e 3, enquanto o Grupo 2 apresentou escores 2 e 3 em cerca de 11% (n = 3) dos dentes. O escore da RRAE aumentou em ambos os grupos após a movimentação ortodôntica; entretanto, as análises estatísticas não mostraram diferenças significativas entre os grupos (*p*> 0,05).

**Conclusões:**

Dentes submetidos à movimentação ortodôntica sobre rebordo edêntulo atrófico estão sujeitos a uma maior reabsorção radicular externa lateral.

## INTRODUCTION

After a tooth is extracted, a dimensional reduction of the alveolar bone occurs. One year later, it can reduce to an average of 50%.^1^ Such dimensional loss is more pronounced on the buccal than on the lingual side of the alveolus, and makes implant placement difficult.^2^ Among the various procedures for improvement of the alveolar ridge, there are several types of grafting surgeries, lateralization and transposition of the inferior alveolar nerve. However, they can be considered invasive and/or expensive. In addition, vertical stability of the grafts, in general, presents poor predictability.^3^ In this context, the use of orthodontic movement becomes an interesting alternative to restore the dimensions of the atrophic ridge, optimizing the relationship between the adjacent hard and soft tissues.^4-7^

To have proper preservation of the alveolar bone as well as root integrity, the movement must take place in the absence of plaque, which can promote additional inflammation.^8^ ^,^ ^9^ The force used should also maintain a physiological level, since excessively heavy forces can result in significant root resorption.^10-12^ Although mild root resorption is considered inherent to orthodontic movement,^13^ ^-^ ^15^ the advancement in knowledge of the effects of root architecture and different orthodontic therapies can help reduce the magnitude of the deleterious effects. Although the literature has extensively explored external root resorption in conventional orthodontic treatments,^13-19^ root response when a tooth is moved towards an atrophic ridge is less studied, especially in regards to lateral root resorption.^7^

Given this, the present study aims to compare the external lateral root resorption (ELRR) and external apical root resorption (EARR) between teeth moved through the atrophic edentulous ridge and those undergoing usual orthodontic movement. The null hypothesis tested was that external root resorption is similar in both groups.

## MATERIAL AND METHODS

This retrospective study was approved by the Human Research and Ethics Committee of the University of Maringá (UEM) (CAAE #0045.0.093.000-11). All patients authorized the use of their records. The sample size was calculated considering a test power of 0.8, alpha of 0.05, with a desired difference of 1, as well as a variation of 1 for the score for each patient. Thus, the sample size should be 18 teeth for each group.

Radiographic records of 22 patients (8 males and 14 females) were evaluated: individuals who had lost at least one first molar (for more than 2 years), with subsequent atrophy of the alveolar bone that prevented the installation of dental implants. Mean age of the sample was 46.22 years old (SD = 8.41), ranging from 31.3 to 47 years old. Patients with systemic diseases, active periodontal disease and/or smoking habit were excluded. Previous orthodontic treatment and graft surgery were also exclusion criteria. Five patients had bilateral atrophic regions, and 17 had unilateral ones. A total of 27 premolars were moved through the atrophic alveolar ridge, composing the experimental group (Group 1), and 27 premolars submitted to conventional orthodontic movement, in the same patients, comprised the control group (Group 2). 

Before and after orthodontic movement images taken from patients’ radiographic records were used. They comprised parasagittal slices from cone-beam computer tomography (CBCT) (16 premolars, from 12 patients), periapical (6 premolars, from 6 patients) and panoramic (5 premolars, from 3 patients). All patients received conventional alignment and leveling orthodontic treatment, starting with 0.014-in NiTi archwire, followed by 0.016-in, 0.018-in and 0.020-in stainless steel archwires (Morelli^®^, SP, Brazil). The movement through the atrophic ridge was carried out with NiTi open coil springs inserted on 0.020-in steel archwire and on subsequent 0.019 x 0.025-in steel archwire in 0.022-in brackets (Abzil-3M^®^, SP, Brazil). Mean orthodontic treatment time was 17.15 months (SD = 6.08), ranging from 8.5 to 30.3 months. The mean movement through atrophic area was 5.98 mm (SD = 1.36), minimum 4.5 mm and maximum 10.2 mm. Ten mandibular left second premolars, 11 mandibular right second premolars, 2 mandibular left first premolars, 1 mandibular right first premolars, 2 maxillary left first premolars and 1 maxillary right first premolar were moved through adjacent atrophic alveolar bone. Control tooth in unilateral case was its homologous tooth on the other side (5 mandibular right second premolars, 6 mandibular left second premolars, 2 mandibular right first premolars, 1 mandibular left first premolar, 2 maxillary right first premolars and 1 maxillary left first premolar), and in bilateral sites the adjacent premolar -not moved into the atrophic bone- was evaluated (5 lower right first premolars and 5 lower left first premolars). Control teeth were anatomically similar to the experimental group.

**Figure 1:**
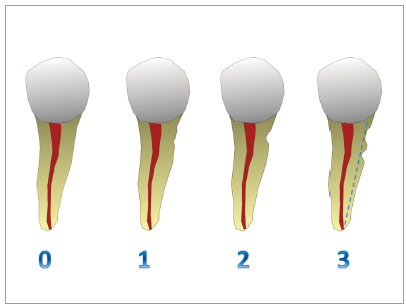
Classification of lateral root resorption: 0 = absence of resorption; 1 = presence of slight resorption lacunae; 2 = presence of a distinct resorption lacunae; 3 = presence of more than one distinct resorption lacunae and/or clear reduction in root thickness.

ELRR was evaluated before and after orthodontic treatment in radiographic records, according to the following scores from 0 to 3: 0 = absence of resorption; 1 = presence of slight resorption lacunae; 2 = presence of a clear resorption lacunae; 3 = presence of more than one distinct resorption lacunae and/or clear reduction in root thickness (Fig. 1).

**Figure 2:**
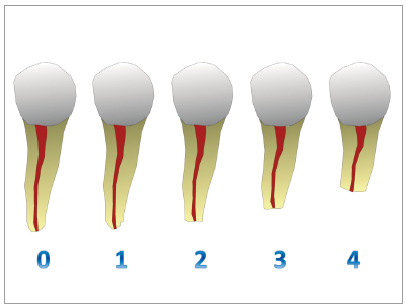
Classification of apical root resorption: 0 = absence of resorption; 1 = mild resorption, irregular apical contour; 2 = moderate resorption, small root loss, with the apex displaying an almost straight contour; 3 = marked resorption, loss of almost ⅓ of the root length; 4 = extreme resorption with loss of more than ⅓ of the root length (Source: modified from Levander and Malmgren^13^).

As a secondary outcome, the EARR was also assessed, before and after orthodontic treatment, following the Levander and Malmgren^13^ method. The scores of 0 to 4 ​​were attributed according to the following classification: 0 = absence of resorption; 1 = mild resorption, irregular apical contour; 2 = moderate resorption, small root loss, with the apex displaying a partially straight contour; 3 = marked resorption, loss of almost ⅓ of the root length; 4 = extreme resorption with loss of more than ⅓ of the root length (Fig. 2).

### Statistical analysis

Two calibrated examiners performed evaluations. Measurements were repeated for all the images after an interval of 30 days. The agreement was checked by Kappa weighted test. External root resorption scores were compared by Kruskal-Wallis, followed by Student-Newman-Keuls post-test, using BioEstat 5.0 software (Instituto Mamirauá, AM, Brazil). 

## RESULTS

Kappa tests showed good agreement between the two moments of evaluation for lateral scores (examiner 1 = 0.82; examiner 2 = 0.80), as for apical scores (examiner 1 = 0.79; examiner 2 = 0.81). Inter-examiner Kappa tests also showed good agreement (0.81 for lateral scores and 0.83 for apical scores). Medians of scores between the two examiners were used for statistic comparisons.

**Figure 3:**
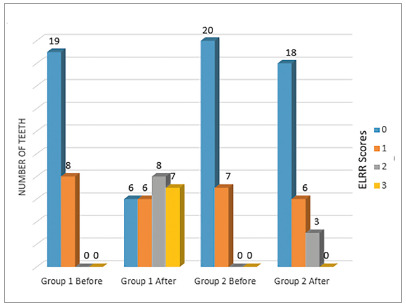
Distribution of teeth in scores related to lateral root resorption (ELRR) before and after orthodontic treatment.

In Kruskal-Wallis/ Student-Newman-Keuls comparison between before and after treatment, ELRR increased statistically only in the Group 1 (Table 1). The teeth moved over the ridge had higher lateral resorption scores. After orthodontic treatment, it was observed that almost 56% (n = 15) of the teeth in Group 1 presented scores 2 and 3 (2 = presence of a clear resorption lacunae; 3 = presence of more than one distinct resorption lacunae and/or clear reduction in root thickness), while Group 2 presented those scores in about 11% (n = 3) of the teeth (Fig. 3).

**Table 1:** Median, 1^st^ quartile and mean scores of the ELRR and Kruskal-Wallis/ Student-Newman-Keuls comparisons.

	Group 1		Group 2	
	Before	After	Before	After
Median (1^st^ - 3^rd^ quartiles)	0 (0-1)^a^	2 (1-3)^b^	0 (0-0.5)^a^	0 (0-0.5)^a^

Different superscript letters represent statistical significance at *p*< 0.05.

**Figure 4:**
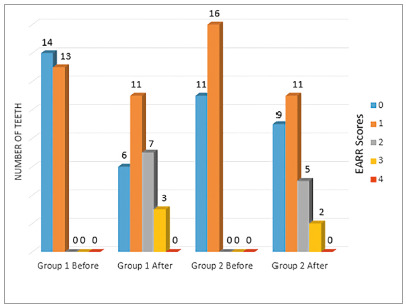
Distribution of teeth in the scores related to apical root resorption (EARR) before and after orthodontic treatment.

EARR increased in both groups after orthodontic movement (Table 2), however, statistical analyses showed no significant differences between groups (*p*> 0.05). The EARR scores distribution is shown in Figure 4.

**Table 2:** Median, 1^st^ and 3^rd^ quartiles scores of the EARR and Kruskal-Wallis/ Student-Newman-Keuls comparisons.

	Group 1		Group 2	
	Before	After	Before	After
Median (1^st^ - 3^rd^ quartiles)	1 (0-1)^a^	1 (1-2)^b^	0 (0-1)^a^	1 (0-1)^b^

Different superscript letters represent statistical significance at *p*< 0.05.

## DISCUSSION

The tooth movement over the atrophic ridge have been demonstrated to be an alternative treatment approach for formation of alveolar bone through the transfer path,^8^ allowing better condition for implant placement.^4^ ^-^ ^7^ Besides its advantages, this movement has been less investigated regarding root resorption, mainly the ELRR, that is a less common side-effect during a conventional orthodontic treatment.^7^

In the present study, the null hypothesis should be partially rejected, once the EARR scores were similar in teeth moved through the atrophic edentulous ridge comparing to controls. However, ELRR scores were significantly different in Group 1. It is important to emphasize that none of the teeth in both groups showed periodontal or endodontic commitment during or after orthodontic movement. 

After movement through atrophic ridge, Group 1 showed scores 2 or 3 in almost 56% (n = 15) of the teeth, while the Group 2 presented those scores in about 11% (n = 3). Similar findings were shown by Diedrich et al,^9^ who reported 40.6% of the 32 premolars moved through the atrophic bone. Lindskog-Stokland et al.^7^ reported that some lateral root resorption is an inevitable occurrence after such orthodontic movement.

After an extraction, a dimensional reduction of the alveolar bone occurs. One year later, it can reduce to an average of 50%,^1^ and the width reduction is greater than the loss of height.^20^ The high risk for ELRR may be correlated to the proximity of buccal and lingual cortical plates in edentulous alveolar ridge, as more periodontal stress during tooth movement can be generated in such a dense bone.^21^

EARR is a well-known side effect of the orthodontic treatment, and the second outcome of the present study confirms that. Both groups presented similar scores of EARR, which are also in agreement with previous studies.^7^ ^,^ ^9^ As the apex is far from the cortical plates, this could explain why root responses were similar for both groups. Despite the risk to occur, either EARR and ELRR appear to cease after treatment.^6^ ^,^ ^7^ ^,^ ^10^ ^,^ ^13^ As such external root resorptions are inflammatory and induced by the orthodontic forces, no endodontic treatment is needed.^10^

It was reported that tooth movement for alveolar bone recovery can also be performed through the invaginations of the maxillary sinus.^22^ If there is more cortical bone adjacent to the movement (invagination area), the reshaping will occur more slowly and the moved tooth possible will present more resorption.^23^ In the present study, only 3 patients had premolars moved through maxillary sinus invagination. One of them presented score 3, one presented score 2, and the other one showed score 0, for ELRR after movement. Limitation of the number of maxillary teeth studied did not allow us to affirm precisely, but seems that the root response is similar to that in mandible, in agreement to Lindskog-Stokland et al.^7^

Imaging tests are essential for diagnosing and monitoring root resorption. If in the first 6 months of treatment noticeable external root resorption is diagnosed, the orthodontic treatment must be done in a slower pace.^10^ CBCT allows for the analysis and visualization of images in full size.^14^ ^,^ ^15^ ^,^ ^17^ However, due to the high cost and radiation exposure, it is less used in routine practice. Periapical and panoramic radiographs are more frequently used, and they proved to be a good diagnostic tool for external root resorption.^14^ ^,^ ^15^ However, they present distortions that must be taken into account when performing metric ratings.^17^ This study used qualitative scores that are less influenced by amplification issues. Levander and Malmgren^13^ scores for EARR were created for periapical radiography evaluation, although they can be applied for any radiographic source. Scores are easy to apply, and they set clinically relevant thresholds of EARR diagnostic.^13^ Similarly, in the present study, we developed scores for ELRR for the same reasons. Kappa tests showed similar intraexaminer and interexaminers agreement, both for EARR and ELRR evaluations. Despite this agreement, there were some limitations due to the different radiographic sources. However, this fact was minimized by using scores for the same tooth.

Clinically, it can be interpreted that the movement through the atrophic ridge is an advantageous strategy.^7^ ^,^ ^22^ ^,^ ^24^ ^-^ ^26^ However, individual evaluation is critical for the treatment options on surgical graft or orthodontic-based bone rebuilding. Future studies with quantitative measurements are suggested using CBCT images. 

## CONCLUSION

Orthodontic movement in an atrophic edentulous ridge is subject to a greater risk of external lateral root resorption.

## References

[1] Schropp L, Wenzel A, Kostopoulos L, Karring T (2003). Bone healing and soft tissue contour changes following single-tooth extraction a clinical and radiographic 12-month prospective study. Int J Period Restor Dentistry.

[2] Araujo M G, Lindhe J (2005). Dimensional ridge alterations following tooth extraction An experimental study in the dog. J Clin Periodontol.

[3] Pommer B, Zechner W, Watzek G, Palmer R, Zorzi AR, Miranda JB (2012). To graft or not to graft? Evidence-based guide to decision making in oral bone graft surgery. Bone grafting..

[4] Arslan SG, Tacir IH, Kama JD (2006). Orthodontic and prosthetic rehabilitation of unilateral free-end edentulous space. Aust Dent J.

[5] Gündüz E, Rodríguez-Torres C, Gahleitner A, Heissenberger G, Bantleon HP (2004). Bone regeneration by bodily tooth movement dental computed tomography examination of a patient. Am J Orthod Dentofacial Orthop.

[6] Zachrisson BU (2005). Bjorn U Zachrisson, DDS, MSD, PhD, on current trends in adult treatment, part 2. Interview by Robert G. Keim. J Clin Orthod.

[7] Lindskog-Stokland B, Wennstrom J L, Nyman S, Thilander B (1993). Orthodontic tooth movement into edentulous areas with reduced bone height An experimental study in the dog. Eur J Orthod.

[8] Lindskog-Stokland B, Hansen K, Ekestubbe A, Wennström JL (2013). Orthodontic tooth movement into edentulous ridge areas -- a case series. Eur J Orthod.

[9] Diedrich PR, Fuhrmann RA, Wehrbein H, Erpenstein H (1996). Distal movement of premolars to provide posterior abutments for missing molars. Am J Orthod Dentofacial Orthop.

[10] Krishnan V, Davidovitch Z (2006). Cellular, molecular, and tissue-level reactions to orthodontic force. Am J Orthod Dentofacial Orthop.

[11] Cattaneo P, Dalstra M, Melsen B (2009). Strains in periodontal ligament and alveolar bone associated with orthodontic tooth movement analysed by finite element. Orthod Craniofac Res.

[12] Sameshima GT, Sinclair PM (2001). Predicting and preventing root resorption part II. Treatment factors. Am J Orthod Dentofacial Orthop.

[13] Levander E, Malmgren O (1988). Evaluation of the risk of root resorption during orthodontic treatment a study of upper incisors. Eur J Orthod.

[14] Freitas JC, Lyra OCP, Alencar AHG, Estrela C (2013). Long-term evaluation of apical root resorption after orthodontic treatment using periapical radiography and cone beam computed tomography. Dental Press J Orthod.

[15] Segal GR, Schiffman PH, Tuncay OC (2004). Meta-analysis of the treatment related factors of external apical root resorption. Orthod Craniofac Res.

[16] Silveira HL, Silveira HE, Liedke GS, Lermen CA, Santos RB, Figueiredo JA (2007). Diagnostic ability of computed tomography to evaluate external root resorption in vitro. Dentomaxillofac Radiol.

[17] Dudic A, Giannopoulou C, Leuzinger M, Kiliaridis S (2009). Detection of apical root resorption after orthodontic treatment by using panoramic radiography and cone-beam computed tomography of super-high resolution. Am J Orthod Dentofacial Orthop.

[18] Årtun J, Smale I, Behbehani F, Doppel D, Van't Hof M, Kuijpers-Jagtman AM (2005). Apical root resorption six and 12 months after initiation of fixed orthodontic appliance therapy. Angle Orthod.

[19] Marques LS, Ramos-Jorge ML, Rey AC, Armond MC, Ruellase ACO (2010). Severe root resorption in orthodontic patients treated with the edgewise method prevalence and predictive factors. Am J Orthod Dentofacial Orthop.

[20] Van der Weijden F, Dell'Acqua F, Slot DE (2009). Alveolar bone dimensional changes of post-extraction sockets in humans a systematic review. J Clin Periodontol.

[21] Horiuchi A, Hotokezaka H, Kobayashi K (1998). Correlation between cortical plate proximity and apical root resorption. Am J Orthod Dentofacial Orthop.

[22] Re S, Cardaropoli D, Corrente G, Abundo R (2001). Bodily tooth movement through the maxillary sinus with implant anchorage for single tooth replacement, Clin Orthod. Res.

[23] Park JH, Tai K, Kanao A, Takagi M (2014). Space closure in the maxillary posterior area through the maxillary sinus Am J Orthod Dentofacial. Orthop.

[24] Ramos AL (2013). An interview with Adilson Luiz Ramos Dental Press J. Orthod.

[25] Rosa JMP, Machado MF, Khoury EMDA, Ramos AL (2012). Prospective study of induced tooth movement as an alternative for bone ridge augmentation. Ortodontia.

[26] Eliášová P, Marek I, Kamínek M (2014). Implant site development in the distal region of the mandible bone formation and its stability over time. Am J Orthod Dentofacial Orthop.

